# The Role of Copigmentation in Colour Attributes and Their Evolution in Model Wine: A Thermodynamic and Colorimetric Study

**DOI:** 10.3390/foods14142467

**Published:** 2025-07-14

**Authors:** Arianna Ricci, Cristian Galaz-Torres, Giuseppina Paola Parpinello, Miriana Demola, Marco Spiga, Andrea Versari

**Affiliations:** 1 Department of Agricultural and Food Sciences (DISTAL), Alma Mater Studiorum-University of Bologna, Piazza Goidanich 60, 47521 Cesena, FC, Italy; arianna.ricci4@unibo.it (A.R.); cristian.galaz2@unibo.it (C.G.-T.); miriana.demola@studio.unibo.it (M.D.); marco.spiga4@studio.unibo.it (M.S.); andrea.versari@unibo.it (A.V.); 2 Inter-Departmental Centre for Agri-Food Industrial Research (CIRI-Agro), Alma Mater Studiorum-University of Bologna, Via Quinto Bucci 336, 47521 Cesena, FC, Italy

**Keywords:** copigmentation, red wine colour, CIELab, anthocyanins, wine ageing

## Abstract

The colour evolution of malvidin-3-*O*-glucoside (Mv-3-*O*-glc) elicited by caffeic acid (CAF), (+)-catechin (CA), or syringic acid (SI) was spectrophotometrically monitored in model wine solution, modulating the malvidin-to-polyphenol molar ratio (1:1 to 1:20) and the pH (2.8–3.8). The spectral features provided the thermodynamic parameters Gibbs free energy (ΔG^0^) and equilibrium constant (*K_eq_*), showing that the copigmentation extent is maximized at pH 3.6 and a higher molar ratio (1:20), and that copigments have different efficiency. In a long-term evolution (12 months), transient complexes evolved into different colour characteristics. Spectrophotometry and colorimetry (chroma C*, hue H*, and lightness L*) revealed the formation of stable pigments with peculiar orange-reddish colour when CAF was present; however, in the case of CA, an accentuated yellow tone was observed. SI showed minimum impact in the long-term evolution of Mv-3-*O*-glc. This study expands knowledge on oenological copigmentation, further exploring its potential implication in the colour of aged red wines.

## 1. Introduction

Many factors are acknowledged to influence the colour of beverages containing anthocyanins. Among these, copigmentation has a strong stabilizing effect in red wines: in its absence, the common anthocyanins exist essentially in their colourless form under oenological conditions. Although the copigmentation mechanism is still partly to be clarified, many studies have provided a valuable theoretical background to tackle the issue, also considering the technological implications in terms of the colour attributes and stability of red wines. As described in the previous literature [1,2,3,4], copigmentation is relevant for the wine industry, affecting the colour intensity and shade of young red wines; at the same time, copigmentation preserves the monomeric anthocyanins from early oxidative phenomena, with a positive effect on the long-term evolution of wine colour.

Copigmentation is an exothermic and spontaneous process commonly observed in nature that involves the stacking of a colourless, organic molecule with an electron-rich π system—the so-called ‘copigment’ or ‘cofactor’—on the electron-poor polarizable moieties of a pigment, i.e., the flavylium form of anthocyanins. Different mechanisms have been postulated to account for the resulting molecular association: (i) a combination of π-π and OH-π interactions with repulsive Coulomb-type interactions, (ii) hydrophobic interactions, and (iii) a charge transfer from the electron-rich form (copigments) to the electron-poor centre of the pigment. The interaction is favoured by the planar structure of the molecules involved which facilitates the stacking [5,6] and besides that, the extent of copigmentation can be dependent upon several factors, including the pigment/copigment molar ratio and the molecular structure/substituents of the couple involved, along with environmental factors including pH and the ionic strength of the medium, solvent, and temperature [1,7]. According to the chemical structure, the copigments are considered stoichiometric reagents including a variety of structurally unrelated colourless compounds, including alkaloids, amino acids, organic acids, and polyphenol/flavonoid compounds, these latter being the most relevant for red wines [1,8].

Copigmentation is a swift dynamic process in solution and takes place in fermenting musts and young red wines as long as free anthocyanin species remain at a sufficient concentration level; the lifetime of the complexes formed can be in the order of microseconds, and the evident colour effect results from several and brief encounters between partners [6,9]. In terms of technological impact, we can hypothesize two scenarios: the first relates to the ‘protective effect’ of the supramolecular associations, which prevents the anthocyanins from oxidation–hydration- and self-association mechanisms without permanently altering the flavylium ion’s basic structure; in this case, the specific copigmentation couple has little to do with the ageing of wine colour. Nevertheless, if the transient complexes formed evolve toward stable molecular structures with chromophore groups, we can consider copigmentation the first molecular step for the development of new pigmented species, according to the hypothesis of Brouillard and Dangles [10], further confirmed by Boulton [1] and, in more recent times, by Dippenaar et al. [11]:

$$\begin{array}{ll} \text{anthocyanin}+\text{copigment} & \overset{FAST}{⇄}\text{copigmentation complex}\\ \text{copigmentation complex} & \overset{SLOW}{⟶}\text{new pigment} \end{array}$$

Preliminary studies [10,12] have suggested that the new pigment could be of xanthylium type, which typically contributes with yellow-orange hues, and it is more resistant toward nucleophile reactions (covalent hydration and bleaching by bisulfite ions) and less susceptible to pH changes than flavylium ions under oenological conditions [13,14]. Nevertheless, as different molecular features provide different copigmentation effects, it is likely to hypothesize a variety of chemical structures with different long-term impacts on the chromatic characteristics of red wines [3].

Despite the fact that there are many studies aimed at modelling the phenomenon of wine copigmentation [15,16,17,18,19], to the best of our knowledge, there is still a need of an integrated theoretical approach considering the evolution of copigmentation couples during storage and under different and oenologically relevant conditions. In this view, this work aims to provide spectrophotometric and colorimetric evidence on how the intermolecular copigmentation elicited by different polyphenolic structures, in different molar ratios with pigments, and under relevant oenological pH values may impact the wine colour characteristics in short- and long-term storage. Caffeic acid (CAF), (+)-catechin (CA), and syringic acid (SI) were selected as representatives of three key polyphenolic subclasses in wine due to their easy availability and wide use in mechanistic studies using model solutions [15,17,18,19,20], which enabled a direct comparison with previous results. The results under standardized conditions will serve as the foundation for future mechanistic studies to describe the dynamic of red wine fining.

## 2. Materials and Methods

### 2.1. Reagents and Experimental Setup

Malvidin-3-*O*-glucoside (Mv-3-*O*-glc, oenin ≥ 95%), the most representative anthocyanin in *Vitis vinifera* L., and three representative polyphenol copigments, i.e., syringic (SI, ≥98%) for benzoic acids, caffeic (CAF, ≥99%) for hydroxycinnamic acids, and (+)-catechin (CA, ≥99%) for flavan-3-ols, were purchased from Extrasynthese (Genay Cedex, France). The chemical structure of standards used in this study is reported in Figure S1. Standards were dissolved in a model wine solution of 12% ethanol *v*/*v* (standard purity > 99%), L-tartaric acid 0.033 M in distilled water (ionic strength adjusted to 0.02 M by KCl addition); all chemicals were from Sigma–Aldrich (Milan, Italy). A few drops of hydrochloric acid 37% and sodium hydroxide from 1 to 10 M were used to adjust the model wine solution at pH 2.8, 3.2, 3.6, and 3.8 using a laboratory HANNA 209 pH meter (Hanna Instruments Inc., Woonsocket, RI, USA). For each couple considered, 0.5 mL of 1 × 10^−4^ M Mv-3-*O*-glc was mixed with an equal volume of copigment solutions at the different concentration levels, to give a final 5 × 10^−5^ M Mv-3-*O*-glc and pigment/copigment molar ratios of 1:1, 1:5, 1:10, and 1:20; the copigmentation couples were labelled Mv-3-*O*-glc/CAF, Mv-3-*O*-glc/CA, and Mv-3-*O*-glc/SI, respectively. The Mv-3-*O*-glc content was kept low to avoid colour interferences due to self-association effects [20].

The study was designed with a two-step experiment:

(1) **Short-term spectrophotometric monitoring:** Short-term spectrophotometric monitoring aimed at determining the magnitude of the copigmentation effect (bathochromic and hyperchromic) by increasing the molar ratio of copigments with constant Mv-3-*O*-glc concentration and changing pH over a range of oenological interest (2.8–3.8). All these solutions were prepared in triplicate and were stored in closed glass vials in the dark at 20 °C for 30 min to reach equilibrium. Experiment 1 allowed us to determine the spectral shift (hyperchromicity) used to calculate the equilibrium constant (*K_eq_*), the Gibbs free energy variation at the equilibrium (ΔG^0^), and the stoichiometry of the copigmentation for every copigment, molar ratio, and pH value considered (details in Section 2.2).
(2) **Long-term spectrophotometric and colorimetric monitoring:** The spectrophotometric and colorimetric analysis of the working solution after 12 months of storage in the dark at room temperature aimed at revealing the colour evolution patterns for the copigmentation couples considered. The solutions were stored in screw-cap glass vials filled to their maximum volume to avoid hyperoxygenation.

### 2.2. Spectrophotometric Analysis

The global extent of the copigmentation can be spectrophotometrically estimated as an increased absorbance intensity at the maximum wavelength (hyperchromic shift) and the shift in the maximum towards higher wavelength (bathochromic) resulting in more pronounced purple hues [21,22]. The spectra were collected with a Cary^®^ 60 UV/Vis Spectrophotometer (Agilent Technologies, Milan, Italy) in dual beam mode, using a 1 cm length-path quartz cuvette. Spectral patterns were recorded over the 300–700 nm visible range, with a scan rate of 600 nm/min and a data interval of 1 nm. Occasional noisy signals were smoothed using a 9-point Savitzky–Golay filter from the Cary WinUV Software version 5.1.0.1016 (Agilent Technologies, Milan, Italy).

According to the theoretical framework proposed by Brouillard and coworkers [21] and consolidated in more recent studies [23], the relationship (A − A_0_)/A_0_ measures the hyperchromic effect occurring around the maximum absorbance of Mv-3-*O*-glc (523 nm) and relates to the magnitude of the molecular interaction in copigmentation. Based on this assumption, hyperchromicity allows researchers to obtain the equilibrium constant of the copigmentation process (Keq, M^−1^) using the following Equation (1):

Ln [(A − A_0_)/A_0_] = ln(*K_eq_*) + n ln [C_p_]_0_ (1)

where

A_0_ (λ = 523 nm): absorbance of Mv-3-*O*-glc 5 × 10^−5^ M;
A (λ = 523 nm): absorbance of Mv-3-*O*-glc 5 × 10^−5^ M after copigment addition;
*K_eq_*: thermodynamic equilibrium constant for the molecular association (copigmentation), M^−1^;
n: pigment/copigments stoichiometry;
[C_p_]_0_: initial copigment concentration.

**Figure 1:**
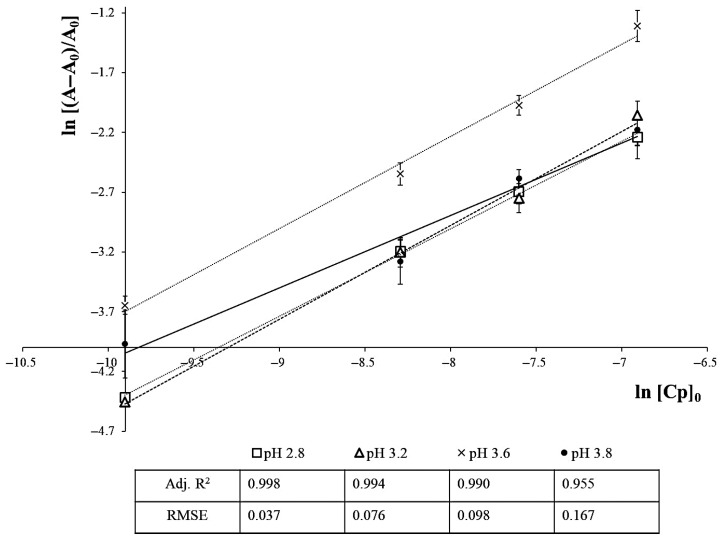
Plot of ln [(A − A_0_)/A_0_] vs. ln [C_p_]_0_ to calculate the n and *K_eq_* parameters of the copigmentation reaction, exemplified for the Mv-3-*O*-glc/CAF couple.

The n and *K_eq_* can be graphically obtained by plotting ln [(A − A_0_)/A_0_] vs. ln [C_p_]_0_ (Figure 1), producing a straight line with n as the slope and ln*K_eq_* as the intercept. The Gibbs free energy variation associated with the process (ΔG^0^, kJ mol^−1^) was obtained by the classic approach for equilibrium calculations in chemical reactions (2):

ΔG^0^ = −RT ln*K_eq_* (2)

where

R: gas constant 8.314 J mol^−1^ K^−1^;
T: temperature (standardized at 298 K).

### 2.3. Colorimetry

The CIELab chromatic coordinates L*, a*, and b*, with illuminant D65 and 10° observer [24], were calculated using the DNAPhone Smart Analysis technology (DNAPhone, Parma, Italy), which also provided the parameters C* (chroma) and H* (hue angle).

### 2.4. Data Processing and Colorimetry Artwork

XLStat-Premium v.2023.1.1 (Addinsoft, New York, NY, USA) was used for data storage, basic statistics, and correlation and to determine the thermodynamic parameters (*K_eq_*, ΔG^0^) and the stoichiometry (n) from the copigmentation reactions. The linear functions of ln [(A − A_0_)/A_0_] vs. ln [Cp]_0_ to calculate the n and *K_eq_* parameters of the copigmentation reaction were assessed by the R^2^ and RMSE regression values. Differences in the Abs 440/510 nm ratio for the pigment formed in the Mv-3-*O*-glc/CA couple after 12 months of storage were studied by means of one-way analysis of variance (ANOVA); the homogeneity of variance assumption for ANOVA was checked using Levene’s test which resulted in a non-significant (>0.05) *p*-value. A Shapiro–Wilk test assessed the null hypothesis with *p* > 0.05, confirming the normal distribution of the data. The Tukey post hoc test was applied when differences were detected among spectral data; both tests were performed at a 5% level of significance (*p*-value < 0.05). Graphical artwork based on the CIELab coordinates was obtained using R-studio^®^ software version 4.2.0 (2022), with the package ggplot2.

## 3. Results

### 3.1. Short-Term Spectrophotometric Monitoring

Table 1 summarizes the results of the spectral processing according to Brouillard et al. [21]. A moderate bathochromic displacement of the λ_523_ nm was observed in all the experiments (range 0.5–2 nm), while the hyperchromic effect was clearly appreciable (Table 1) and informative on the extent of copigmentation; accordingly, the visible spectra (available as metadata) were processed to extrapolate the [(A − A_0_)/A_0_] parameter.

The thermodynamic parameters of the copigmentation onset (Table 1) disclosed a (similar) increased hyperchromicity when increasing copigment molar ratios were mixed with constant Mv-3-*O*-glc concentrations, suggesting that the concentration of the copigment is a limiting factor in copigmentation reactions. The effect was confirmed in all experiments regardless of the cofactor and pH values considered. Figure 1 exemplifies the graphical method applied to calculate the equilibrium constant (*K_eq_*) and stoichiometry (n) of Mv-3-*O*-glc/CAF. For every copigment, concentration, and pH value, a slope (n) close to unity was found (Table 1), confirming the pigment/copigment stoichiometry within the range 0.5–2 previously reported by Trouillas et al. [22].

**Figure 2:**
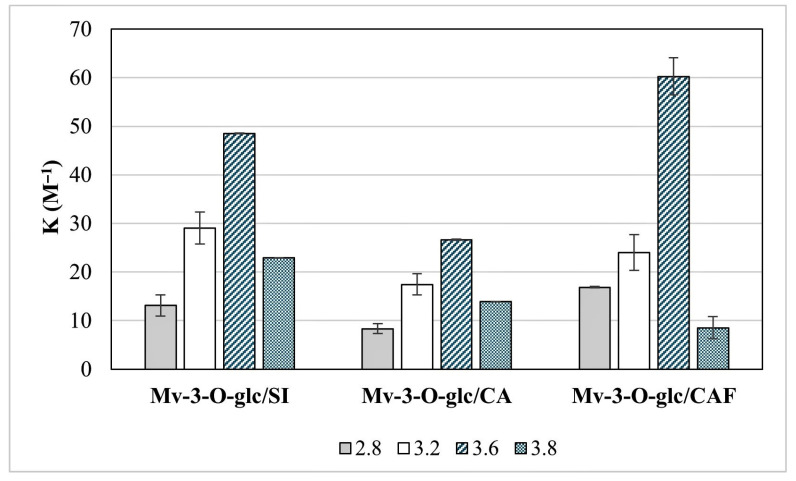
Evolution of the equilibrium constant (*K_eq_*) for the copigmentation of the Mv-3-*O*-glc/SI, Mv-3-*O*-glc/CA, and Mv-3-*O*-glc/CAF couples under different pH conditions.

The thermodynamic values reported in Table 1 and Figure 2 reveal that the stability of pigment/copigment association has a strong dependence on the pH, being more favourable at 3.6 (higher *K_eq_*; Figure 2) for all the molar ratios examined. All copigments followed a similar trend, with a progressive increase in the *K_eq_* value in the pH range 2.8–3.6; the drop in the *K_eq_* value of Mv-3-*O*-glc/CAF (3.2) is probably random according to the standard deviation values (Figure 2). On the contrary, a systematic decrease in the equilibrium constants observed from 3.6 to 3.8 suggests that increasing pH values may negatively affect the occurrence and stability of copigmentation complexes. As further confirmation, copigmentation was thermodynamically favoured at pH 3.6 according to the Gibbs free energies at equilibrium (lower ΔG^0^) for all copigments, and the process’s energy level increased at a pH value of 3.8 (Table 1). At the same time, all ΔG^0^ are negative (Table 1), informing that the copigmentation always proceeds spontaneously, although to a different extent, under our experimental conditions.

According to the thermodynamic parameters of the standard solutions (Table 1), the strength of the copigmentation couples ranked Mv-3-*O*-glc/CAF > Mv-3-*O*-glc/SI > Mv-3-*O*-glc/CA in our experiment. The *K_eq_* found for the couple Mv-3-*O*-glc/CAF was 65.0 ± 9.1 M^−1^, confirming CAF as the strongest copigment; the hyperchromicity was more pronounced with an increasing molar ratio of CAF, until it reached a maximum for the pigment/copigment 1:20 (Table 1).

The maximum strength for the Mv-3-*O*-glc/CA copigmentation complex was found at pH 3.6 and resulted in *K_eq_* = 26.6 M^−1^ and ΔG^0^ = −7.89 kJ mol^−1^ (Table 1); the same pH value was confirmed to enhance the copigmentation for the Mv-3-*O*-glc/SI couple, with higher *K_eq_* (48.5 M^−1^) and ΔG^0^ (−9.56 kJ mol^−1^) values.

### 3.2. Long-Term Spectrophotometric Monitoring

The CIELab colour parameters of the model solutions after 12 months of storage were compared with Mv-3-*O*-glc solutions with different pH at time zero (Control T0), and results are reported in Table 2 as CIELab parameters supported by a visual colour rendering. Concerning Mv-3-*O*-glc/CAF (Table 2a), a notable difference in the colour profile, i.e., higher C* and lower L*, was observed between pH 3.2 and 3.8, and was particularly pronounced at pH 3.6; on the contrary, limited differences in H* were generally observed. An orange-ish hue was observed with an increase in C* and a decrease in L* corresponding to a decreasing molar ratio, with limited impact on H*; as for the influence of pH, the colour characteristics of the treatments became increasingly homogeneous as pH increased, with a reduction in the total colour C* and higher lightness L*, without affecting the hue H*.

Regarding the CIELab colour parameters of the Mv-3-*O*-glc/CA treatments after 12 months (Table 2b), a yellowish hue is observed in all molar ratios and pH, with an increase in C* and a slight increase in H*, more marked for the molar ratio 1:1, but without major differences in L*. As for the influence of pH, no major effect was observed except for an increase in L* at pH 3.8 for all molar ratios. On the other hand, comparing the Control T0 with all the solutions after 12 months, the differences in C* and L* became smaller at higher pH, but a strong difference in H* was always maintained for all molar ratios.

When focusing on the Mv-3-*O*-glc/SI treatments after 12 months (Table 2c), a pinkish hue was observed in all molar ratios and pH, without major differences in C*, L*, or H* between molar ratios. As for the influence of pH, no major effect was observed except for an increase in L* at pH 3.8 for all molar ratios, which confirmed the reduced absorbance observed in spectrophotometry (Figure S2).

**Figure 3:**
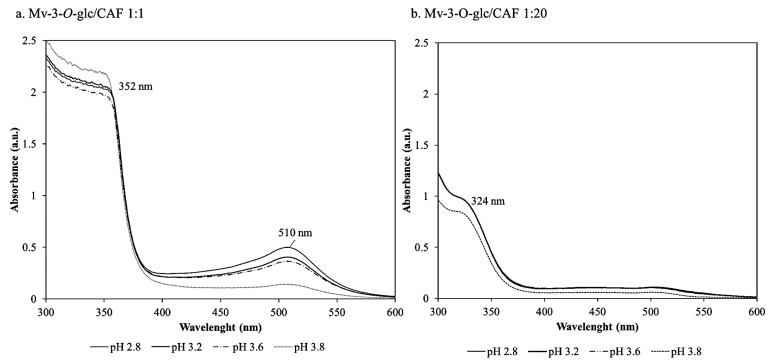
Spectra of the Mv-3-*O*-glc/CAF copigmentation at lower (**a**) and higher (**b**) molar concentrations of CAF after 12 months of storage.

After 12 months of storage, the spectra associated with the Mv-3-*O*-glc/CAF solutions (Figure 3a) showed relevant absorption bands at 510 nm and a shoulder around 400–450 nm that account for the orange hue, along with a second, marked shoulder in the 350–370 nm region. In this experiment, regardless of the absolute absorbance intensities, the spectral profiles overlapped in the pH range 2.8–3.6, while the pH 3.8 spectral profile resembled an oxidized xanthylium structure from Mv-3-*O*-glc. More noticeably, a dependence of the colour evolution on the molar ratio between Mv-3-*O*-glc and the CAF ratio was observed; moving to higher copigment concentrations, the spectral profile changes (Figure 3b), with the prevalence of bands that can be assigned to the unassociated caffeic acid in solution (main absorbance at 324 nm) and to the oxidation derivatives of malvidin (xanthylium ion, 440 nm).

**Figure 4:**
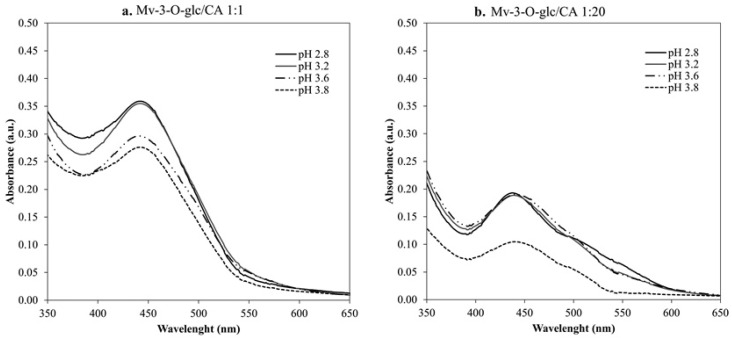
Spectra of the Mv-3-*O*-glc/CA copigmentation at lower (**a**) and higher (**b**) molar concentrations of CA after 12 months of storage.

Evidence of spectral features related to the flavylium/catechin couple was observed in the spectrum recorder after 12 months, with a maximum around 440 to 450 nm, correlated with xanthylium ion-like structures, and a shoulder around 510 nm, contributed by flavylium (Figure 4a,b). The pH values affected the absorbance intensities without altering the spectral profile under the same Mv-3-*O*-glc/CA molar ratio. On the contrary, the molar ratio of the copigmentation couple determined different colour features; the addition of excess CA induced a progressive reduction in the 440/510 nm ratio, making the red component more relevant in Mv-3-*O*-glc/CA 1:20 compared to the other molar ratios investigated (Table 3).

Solutions from the couple Mv-3-*O*-glc/SI produced a spectral profile that resembled the oxidative pattern of Mv-3-*O*-glc in the visible range after 12 months of storage, regardless of the pH value and the molar ratio with malvidin (Figure S2).

## 4. Discussion

In 1999, Mirabel et al. published a pioneering study on the copigmentation of Mv-3-*O*-glc in model wine solution, highlighting its relation to colour evolution during wine ageing [25]. In the same study, the authors recalled that several combinations of coloured and colourless polyphenols are envisaged in red wine, with pigments typically in the range 350–1100 mg/L (7.0 × 10^−4^ M to 7.0 × 10^−3^ M expressed in Mv-3-*O*-glc equivalents), and polyphenols in the range 800 to 4000 mg/L (2.70 × 10^−3^ M to 1.37 × 10^−2^ M in CA molar equivalents): roughly 1:1 to 1:20 total pigment/total copigment molar ratios were reported [25], corresponding to the stoichiometric complexes applied in this study.

The pH-dependent thermodynamic parameters of the copigmentation complexes (Table 1 and Figure 1 and Figure 2) were aligned with the previous findings from the literature [1,26,27], and the systematic reduction in the *K_eq_* at pH value > 3.6 confirmed that copigmentation becomes less relevant, being almost imperceptible approaching pH 4.0 [28]. This is not a trivial aspect and deserves further investigation considering the impact of changing climate conditions (acidity loss and rising pH values) on wine production [29].

Regarding the structure-related copigmentation effect, it is well-established from the literature that copigmentation efficiency depends on the displacement of the hydration equilibrium of anthocyanins, whose entity is affected by the molecular structure and geometry of the cofactors [22]. Caffeic acid (CAF) is an hydroxycinnamic acid typically present at low concentrations in grapes/musts following caftaric acid hydrolysis, promoted by the sunlight exposure of grapes or by enzymatic activity in musts [30,31]. As a hydroxycinnamic acid, it belongs to the C6-C3 phenolic compounds with an acrylic acid side chain, an extended π-conjugated system, and a planar structure, which facilitate π-π stacking. Several studies have demonstrated the strong copigmentation activity of CAF towards glycosylated anthocyanins, both in model-like solutions and real wines [15,23,28,32,33]. The *K_eq_* of the Mv-3-*O*-glc/CAF system was comparable to previous reports under similar experimental conditions (*K_eq_* = 70 ± 20 M^−1^ [15]; *K_eq_* = 74.56 M^−1^ [33]), and so was the ΔG^0^ (−10.4 kJ mol^−1^ vs. −10.7 kJ mol^−1^ after Lambert et al. [15] and −10.5 kJ mol^−1^ after Zhang et al. [33]). Moreover, our work suggested that the effect is more evident when a 1:20 molar ratio is applied. It follows that both colour intensity and hue can be positively influenced by a high CAF concentration, suggesting the role of the hydroxycinnamic acids in the copigmentation of young red wine.

Despite the fact that CAF is generally present in low concentration compared to anthocyanins in wines, its cofactor role can be addressed through pre-fermentative addition strategies as suggested in the recent literature [34,35,36]. In addition to colour stabilization, the Mv-3-*O*-glc/CAF complex was found to exhibit enhanced reactivity (related to both electron and hydrogen transfer) compared to Mv-3-*O*-glc alone [37,38], with a potential active role in the antioxidant protection of wines.

(+)-Catechin (CA) is one of the main flavan-3-ol compounds in red grapes, being involved in antiradical and condensation reactions, and responsible for the oxidative formation of quinones. The *K_eq_* and ΔG^0^ values found in our study (Section 3.1 and Table 1) were lower compared to previous results (pH 3.5 *K_eq_* = 136 ± 4 M^−1^, computational [2]; pH 3.6 *K_eq_* = 90 ± 20 M^−1^ and ΔG^0^ = −11.2 kJ mol^−1^, experimental [15]), suggesting the low stability and reproducibility of the transient complexes. Nevertheless, our findings confirmed the relatively weak cofactor efficiency of CA due to the nonplanar structure of the electron-rich centre of the molecule (ring C) that discourages the π-π stacking [6,8,25,39]. The relevance of the CAF-driven copigmentation compared to CA in short-term colour evolution was confirmed in previous experiments involving the pre-fermentative addition of the two cofactors in winemaking [40]. On the other hand, the long-term monitoring of the CAF- and CA-based complexes provided further insight into their role during wine ageing.

The experiment with syringic acid (SI), a benzoic acid representative, produced intermediate performances in terms of copigmentation efficiency. The thermodynamic values obtained at pH 3.6 for the Mv-3-*O*-glc/SI couple reproduced well the previous findings of Malaj et al. [27], with *K_eq_* = 48.12 M^−1^ and ΔG^0^ = −9.60 kJ mol^−1^ under pH 3.65, despite the different solvent used (12% *v*/*v* ethanol compared to 8% v/v methanol). The same authors [27] found that SI was the best performant hydroxybenzoic acid copigment owing to its di-*O*-methylated molecular structure. The ranking of copigmentation efficiency of polyphenols according to their methylation patterns was confirmed by independent authors [27,41,42], leading to the following assumption: in wine, non-methylated (i.e., gallic acid) are the prevalent hydroxybenzoic acid forms, but copigmentation may be largely contributed by minor *O*-methylated compounds (i.e., vanillic and syringic acid).

The spectrophotometric and colorimetric analyses of the working solutions after 12 months of storage in the dark revealed the different evolution of the pigment/copigment couples.

The spectral and colorimetric features observed for Mv-3-*O*-glc/CAF resembled typical pyrano-anthocyanin-phenol structures that result from direct reaction between anthocyanins and hydroxycinnamic acids [3,43]. The literature confirms our findings in relation to the orange-red colour hue, the higher extinction coefficient compared to the anthocyanins from which they originate, and the relative stability against pH modifications [3,44]. Our results suggested a relevant role for CAF in ensuring colour stability: (i) producing a strong copigmentation effect in short-term monitoring and (ii) promoting the formation of highly stable pigments with a strong colouring effect during the long-term ageing of red wines.

Regardless of the limited role of CA in copigmentation, the flavan-3-ol was relevant in wine colour evolution during ageing. The spectral profiles reported for the Mv-3-*O*-glc/CA couple after 12 months (Figure 4a,b) suggested the occurrence of a coupling reaction, as previously observed by Escribano-Bailòn et al. [45]. Moreover, the pH affected the absorbance intensity but not the spectral profile under the same Mv-3-*O*-glc/CA molar ratio, suggesting that the newly formed pigments can undergo pH-dependent fading (hydration) which affects the colour intensity without altering the colour hues. Early studies [45] have demonstrated that coupling reactions between the flavan-3-ol and the flavylium ion may occur following the copigmentation of the Mv-3-*O*-glc/CA pair. The mechanism was reported as flavylium/catechin copigmentation, followed by C8-C8 covalent coupling to form a dimer; π-π stacking is a first step to bring the molecules closer, making it more likely for the covalent reaction to occur. At the same time, the evolution of CA into a xanthylium form is envisaged during ageing, and the colour expression of the xanthylium pigment preferentially involves non-covalent dimerization in solution due to its chemical structure, with minor or no contribution from the vertical stacking [19].

Although the results of this experiment confirmed CA as a weak copigment and potential browning factor, the evolution of the 440/510 nm reported in Table 3 suggests that the molar ratio can be a limiting factor for the evolution of colour characteristics, and the low propensity to copigmentation can be offset by increasing the molar contribution from CA- and CA-based compounds up to Mv-3-*O*-glc/CA 1:10 to maximize the reddish hues; in this regards, the addition of condensed oenological tannins can be a priority strategy to preserve wines from undesirable colour tunings.

Considering the long-term colour evolution of the Mv-3-*O*-glc/SI couple, results from Table 2c and Figure S2 recall that the copigmentation onset in the short-term storage experiment has little to do with the long-term evolution of the colour; most likely, the transient species formed at the early stage of the experiment shortly reversed their structure, releasing free Mv-3-*O*-glc in solution. The result confirms previous findings that syringic and, more generally, hydroxybenzoic acids (aggregate Σ) are not significant contributors to the chemical processes of wine ageing [46,47].

## 5. Conclusions

In summary, the interaction between Mv-3-*O*-glc, the main pigment in red wines, and polyphenolic compounds typically found in red wines was investigated using spectrophotometry, thermodynamic, and colorimetric approaches. CAF exhibited a larger short-term copigmentation extent with Mv-3-*O*-glc, which also constituted a prerequisite in the development of stable pigments during long-term storage. The low copigmentation activity of CA was confirmed under all simulated oenological conditions, while its presence can promote the formation of yellowish colours due to the prevalence of xanthylium-type structures during ageing. The presence of SI had a major impact in the copigmentation process but no contribution to colour characteristics in the long term. In a way, our study provides a theoretical basis and suggests technical strategies (acidification and deacidification; the pre-fermentative addition of copigments) to promote the quality attributes of red wines. Further studies are envisaged to evaluate the dynamic of the different copigmentation couples and reveal their role in the complexity of wine ageing mechanisms.

## Figures and Tables

**Table 1:** Spectrophotometric evaluation of the hyperchromic shift (λmax = 523 nm) due to the copigmentation of Mv-3-*O*-glc with caffeic acid (Mv-3-*O*-glc/CAF), (+)-catechin (Mv-3-*O*-glc/CA), and syringic acid (Mv-3-*O*-glc/SI), and thermodynamic parameters derived according to Brouillard et al. [21].

Pigment/Copigment Couple	pH	Hyperchromic Shift (A − A_0_)/A_0_ (λ_523nm_)				r^2^	Stoichiometry	ΔG^0^
		1:1	1:5	1:10	1:20		(n)	(kJ mol^−1^)
Mv-3-*O*-glc/CAF	2.8	0.0097	0.0326	0.0568	0.0906	0.999	0.76	−7.05
	3.2	0.0174	0.0640	0.0814	0.1628	0.985	0.74	−7.92
	3.6	0.0217	0.0696	0.1304	0.2435	0.996	0.85	−10.40
	3.8	0.0189	0.0472	0.0755	0.1226	0.982	0.63	−5.31
Mv-3-*O*-glc/CA	2.8	0.0152	0.0455	0.0732	0.0972	0.996	0.64	−5.26
	3.2	0.0197	0.0590	0.0927	0.1573	0.996	0.69	−7.12
	3.6	0.0435	0.1304	0.2043	0.2826	0.991	0.64	−7.89
	3.8	0.0189	0.0566	0.0755	0.1509	0.987	0.67	−6.56
Mv-3-*O*-glc/SI	2.8	0.0214	0.0671	0.1031	0.1425	0.991	0.65	−6.38
	3.2	0.0184	0.0642	0.1055	0.1683	0.998	0.75	−8.38
	3.6	0.0294	0.1131	0.1819	0.2593	0.988	0.76	−9.56
	3.8	0.0189	0.0736	0.1129	0.1501	0.978	0.71	−7.77

**Table 2:** CIELab parameters of the copigmentation couples at different pH values, compared to the initial Mv-3-*O*-glc solutions (Control T0). The table includes a colour rendering according to the CIELab coordinates.

**a: Mv-3-*O*-glc/CAF**									
**pH**	**Treatment**	**Time**	**Molar Ratio**	C*	H*	L*	a*	b*	**Visible colour**
2.8	Mv-3-*O*-glc	T0		29.29	−1.12	84.44	29.29	−0.57	
3.2	Mv-3-*O*-glc	T0		12.84	0.09	91.31	12.84	0.02	
3.6	Mv-3-*O*-glc	T0		7.56	15.10	93.75	7.30	1.97	
3.8	Mv-3-*O*-glc	T0		3.41	18.13	96.98	3.24	1.06	
2.8	Mv-3-*O*-glc3/CAF	T12	1:1	29.79	31.51	86.40	25.44	15.50	
2.8	Mv-3-*O*-glc3/CAF	T12	1:5	24.84	31.51	89.10	21.20	12.95	
2.8	Mv-3-*O*-glc3/CAF	T12	1:10	18.20	31.51	91.50	15.48	9.58	
2.8	Mv-3-*O*-glc3/CAF	T12	1:20	8.15	39.53	95.50	6.28	5.20	
3.2	Mv-3-*O*-glc3/CAF	T12	1:1	24.19	28.07	88.50	21.33	11.42	
3.2	Mv-3-*O*-glc3/CAF	T12	1:5	20.89	29.79	90.30	18.14	10.36	
3.2	Mv-3-*O*-glc3/CAF	T12	1:10	16.27	30.94	92.20	13.94	8.38	
3.2	Mv-3-*O*-glc3/CAF	T12	1:20	7.57	42.40	95.40	5.60	5.09	
3.6	Mv-3-*O*-glc3/CAF	T12	1:1	22.51	28.07	89.20	19.91	10.51	
3.6	Mv-3-*O*-glc3/CAF	T12	1:5	19.64	29.22	90.70	17.10	9.66	
3.6	Mv-3-*O*-glc3/CAF	T12	1:10	14.38	31.51	89.30	12.26	7.51	
3.6	Mv-3-*O*-glc3/CAF	T12	1:20	7.69	42.40	95.90	5.65	5.21	
3.8	Mv-3-*O*-glc3/CAF	T12	1:1	10.69	37.24	95.80	8.49	6.50	
3.8	Mv-3-*O*-glc3/CAF	T12	1:5	9.44	35.52	96.30	7.68	5.49	
3.8	Mv-3-*O*-glc3/CAF	T12	1:10	9.21	35.52	96.70	7.47	5.38	
3.8	Mv-3-*O*-glc3/CAF	T12	1:20	4.78	48.13	98.50	3.20	3.55	
**b: Mv-3-*O*-glc/CA**									
**pH**	**Treatment**	**Time**	**Molar Ratio**	C*	H*	L*	a*	b*	**Visible colour**
2.8	Mv-3-*O*-glc	T0		29.29	−1.12	84.44	29.29	−0.57	
3.2	Mv-3-*O*-glc	T0		12.84	0.09	91.31	12.84	0.02	
3.6	Mv-3-*O*-glc	T0		7.56	15.10	93.75	7.30	1.97	
3.8	Mv-3-*O*-glc	T0		3.41	18.13	96.98	3.24	1.06	
2.8	Mv-3-*O*-glc3/CA	T12	1:1	24.54	78.50	93.50	4.79	24.07	
2.8	Mv-3-*O*-glc3/CA	T12	1:5	18.46	76.20	93.80	4.47	17.91	
2.8	Mv-3-*O*-glc3/CA	T12	1:10	15.35	75.06	94.30	3.95	14.83	
2.8	Mv-3-*O*-glc3/CA	T12	1:20	12.83	73.34	95.30	3.73	12.28	
3.2	Mv-3-*O*-glc3/CA	T12	1:1	22.71	83.65	94.20	2.47	22.58	
3.2	Mv-3-*O*-glc3/CA	T12	1:5	20.15	84.80	94.70	1.86	20.07	
3.2	Mv-3-*O*-glc3/CA	T12	1:10	13.09	77.92	95.50	2.76	12.79	
3.2	Mv-3-*O*-glc3/CA	T12	1:20	5.79	55.58	96.20	3.28	4.77	
3.6	Mv-3-*O*-glc3/CA	T12	1:1	26.86	84.22	93.50	2.69	26.72	
3.6	Mv-3-*O*-glc3/CA	T12	1:5	23.02	85.37	94.10	1.83	22.95	
3.6	Mv-3-*O*-glc3/CA	T12	1:10	20.27	85.94	95.30	1.48	20.22	
3.6	Mv-3-*O*-glc3/CA	T12	1:20	13.74	79.07	95.60	2.61	13.49	
3.8	Mv-3-*O*-glc3/CA	T12	1:1	22.87	89.38	96.20	0.31	22.87	
3.8	Mv-3-*O*-glc3/CA	T12	1:5	16.42	92.25	97.70	−0.63	16.41	
3.8	Mv-3-*O*-glc3/CA	T12	1:10	11.97	92.25	98.40	−0.45	11.96	
3.8	Mv-3-*O*-glc3/CA	T12	1:20	7.62	85.94	98.30	0.55	7.60	
**c: Mv-3-*O*-glc/SI**									
**pH**	**Treatment**	**Time**	**Molar Ratio**	C*	H*	L*	a*	b*	**Visible colour**
2.8	Mv-3-*O*-glc	T0		29.29	−1.12	84.44	29.29	−0.57	
3.2	Mv-3-*O*-glc	T0		12.84	0.09	91.31	12.84	0.02	
3.6	Mv-3-*O*-glc	T0		7.56	15.10	93.75	7.30	1.97	
3.8	Mv-3-*O*-glc	T0		3.41	18.13	96.98	3.24	1.06	
2.8	Mv-3-*O*-glc3/SI	T12	1:1	7.11	43.54	95.60	5.14	4.91	
2.8	Mv-3-*O*-glc3/SI	T12	1:5	6.81	45.26	96.00	4.80	4.83	
2.8	Mv-3-*O*-glc3/SI	T12	1:10	7.30	46.98	96.00	4.98	5.33	
2.8	Mv-3-*O*-glc3/SI	T12	1:20	6.98	46.41	95.90	4.80	5.08	
3.2	Mv-3-*O*-glc3/SI	T12	1:1	6.15	57.87	96.50	3.29	5.19	
3.2	Mv-3-*O*-glc3/SI	T12	1:5	5.97	57.30	96.40	3.25	5.01	
3.2	Mv-3-*O*-glc3/SI	T12	1:10	6.23	55.00	96.00	3.58	5.10	
3.2	Mv-3-*O*-glc3/SI	T12	1:20	6.12	56.72	96.30	3.37	5.10	
3.6	Mv-3-*O*-glc3/SI	T12	1:1	6.11	54.43	96.20	3.53	4.98	
3.6	Mv-3-*O*-glc3/SI	T12	1:5	5.84	55.58	96.20	3.32	4.81	
3.6	Mv-3-*O*-glc3/SI	T12	1:10	6.20	52.14	95.80	3.79	4.91	
3.6	Mv-3-*O*-glc3/SI	T12	1:20	6.70	53.86	96.20	3.93	5.42	
3.8	Mv-3-*O*-glc3/SI	T12	1:1	3.64	67.04	99.00	1.43	3.35	
3.8	Mv-3-*O*-glc3/SI	T12	1:5	3.45	67.61	99.60	1.30	3.19	
3.8	Mv-3-*O*-glc3/SI	T12	1:10	3.70	65.89	99.00	1.53	3.37	
3.8	Mv-3-*O*-glc3/SI	T12	1:20	3.43	63.03	98.80	1.54	3.06	

**Table 3:** The Abs 440/510 nm ratio for the pigment formed in the Mv-3-*O*-glc/CA couple after 12 months of storage. Results are reported as average values ± SD. ^abc^ Different superscript letters indicate a significant difference among the means in each row; *p* < 0.05 by Tukey’s post hoc test.

	440/510 nm			
	molar 1:1	molar 1:5	molar 1:10	molar 1:20
pH 2.8	2.29 ± 0.01 ^b^	2.40 ± 0.01 ^c^	2.39 ± 0.01 ^c^	1.89 ± 0 ^a^
pH 3.2	2.28 ± 0.01 ^b^	2.40 ± 0.02 ^c^	2.40 ± 0 ^c^	1.91 ± 0.05 ^a^
pH 3.6	2.28 ± 0.01 ^b^	2.39 ± 0 ^c^	2.39 ± 0.01 ^c^	1.89 ± 0.01 ^a^
pH 3.8	2.29 ± 0 ^b^	2.4 ± 0 ^c^	2.40 ± 0.03 ^c^	1.90 ± 0.02 ^a^

## Data Availability

The original contributions presented in the study are included in the article/Supplementary Materials, further inquiries can be directed to the corresponding author.

## Supplementary Materials

The following supporting information can be downloaded at: https://www.mdpi.com/article/10.3390/foods14142467/s1, Figure S1: Chemical structures of the standard polyphenols involved in this study; Figure S2: Spectral profiles of the copigmentation experiment for the couple Mv-3-*O*-glc/SI after 12 months of storage.
