# Supplementary material for: The Role of Copigmentation in Colour Attributes and Their Evolution in Model Wine: A Thermodynamic and Colorimetric Study
Source: Foods. 2025 Jul 14;14(14):2467. doi: 10.3390/foods14142467 (PMC12294291; doi:10.3390/foods14142467)
Supplement: Supplementary file 1 [file foods-14-02467-s001.zip › Supplementary Material Figure S1.pdf]

Supplementary Material Figure S1

Chemical structures of the standard polyphenols involved in this study

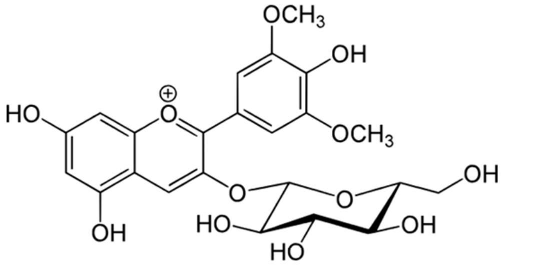


Malvidin-3-O-glucoside (Mv-3-O-glc)

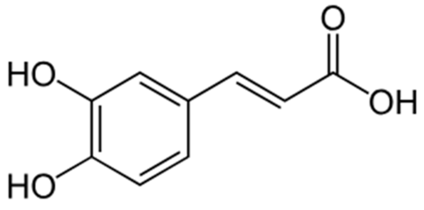


Caffeic acid (CAF)

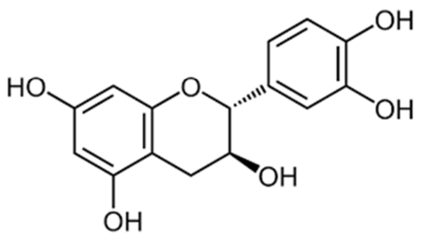


(+)-Catechin (CA)

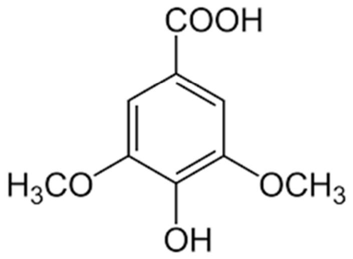


Syringic acid (SI)
