# Supplementary material for: The Role of Copigmentation in Colour Attributes and Their Evolution in Model Wine: A Thermodynamic and Colorimetric Study
Source: Foods. 2025 Jul 14;14(14):2467. doi: 10.3390/foods14142467 (PMC12294291; doi:10.3390/foods14142467)

Supplementary Material Figure S2

Spectral profiles of the copigmentation experiment for the couple Mv-3-O-glc/SI after 12 months storage.

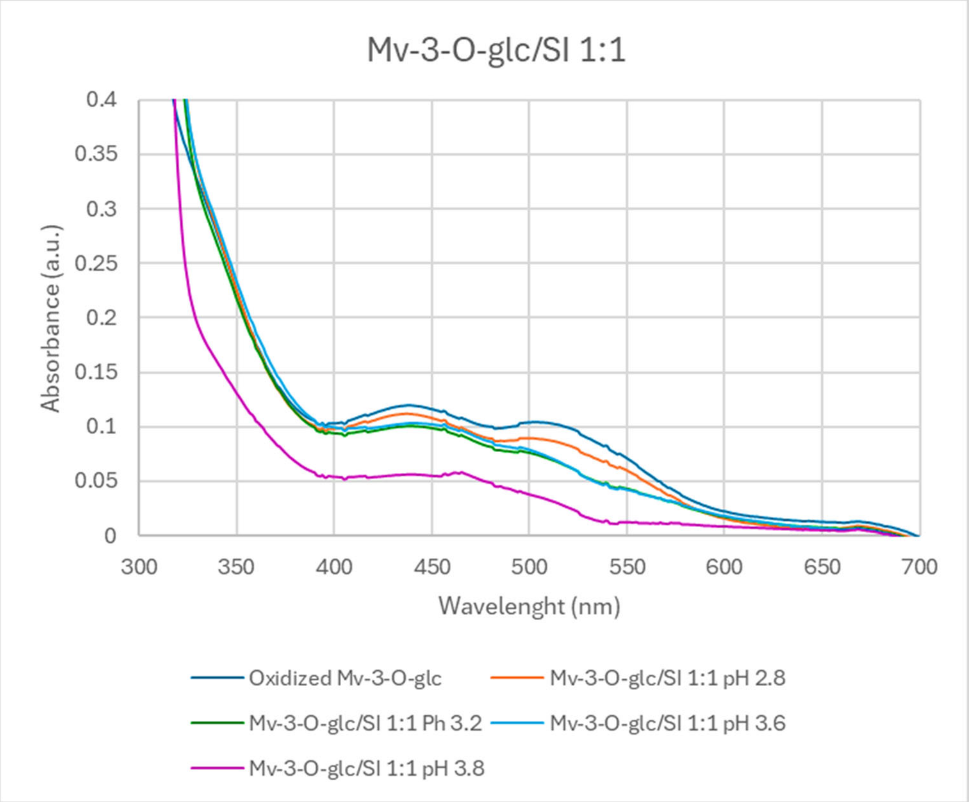

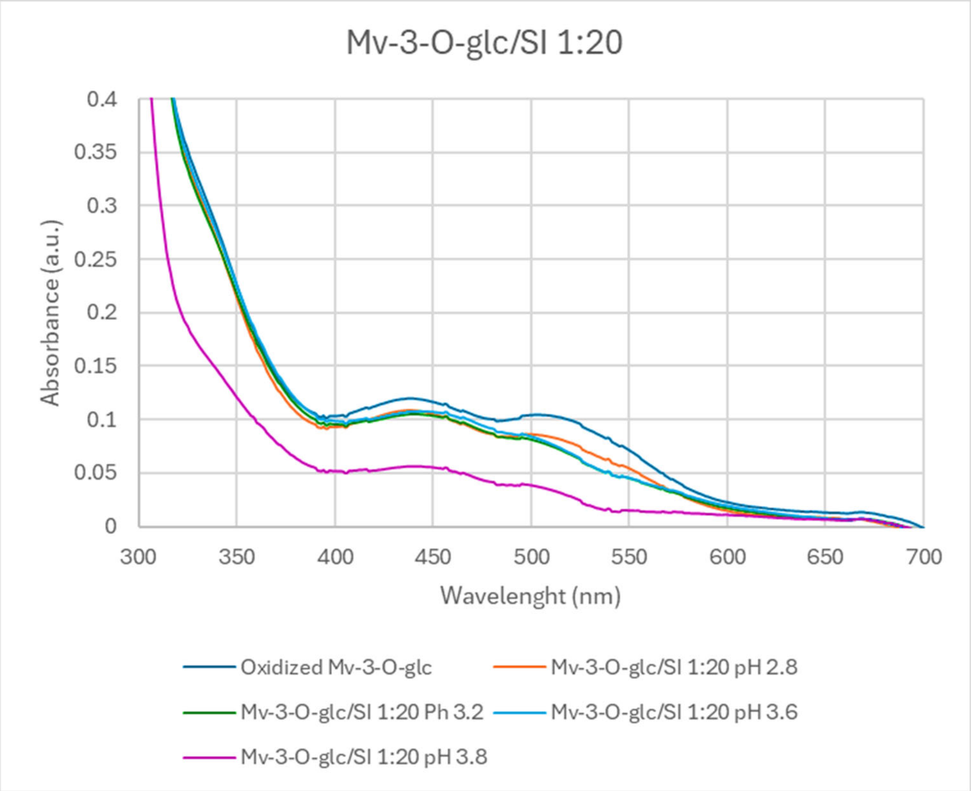

Supplement: Supplementary file 1 [file foods-14-02467-s001.zip › Supplementary Material Figure S2.pdf]
